# Asthma

**Published:** 1898-10

**Authors:** 


Asthma.—The celebrated "*Cigarettes d'Espic*" are said to be made of the following ingredients:

| | | |
|---|---|---|
| ℞ | Belladonna leaves | 5½ parts. |
| | Hyoscyamus leaves | 2¾ parts |
| | Stramonium leaves | 2¾ parts. |
| | Phellandrium aquaticum | 1 part. |
| | Extract of opium (aqueous) | ⅛ part. |
| | Cherry laurel water | q. s. |

The dried leaves, stripped of their stems, are cut small, well mixed and then moistened with the extract of opium dissolved in the cherry laurel water. The paper used for making the cigarettes is also soaked in an infusion of these leaves in cherry laurel water. Usually in making these cigarettes, a little nitrate of potash is added to the infusion to make them burn freely.

The *Carton Fumigatoire* of the French codex, a very useful preparation, is thus made: Take 7 ounces of gray unsized paper and 2 ounces of powdered nitrate of potash; take of belladonna, stramonium, digitalis and lobelia leaves, each 75 grains; take of powdered myrrh and olibanium, each 150 grains. Tear the paper in pieces and soak it in water, then add the powders previously mixed, and pound and beat them all together. Then spread out the soft paste in tin moulds and dry it in a stove. Finally, cut this quantity into 36 pieces, each 6 cm. long and 4 cm. wide. One of these is burned in the patient's room.

The following is given as Himrod's cure:

| | | |
|---|---|---|
| ℞ | Lobelia leaves powdered | aa ℥j |
| | Black tea leaves powdered | |
| | Stramonium leaves powdered | |

Pour upon this mixture 2 ounces of a saturated solution of nitrate of potash, mix thoroughly, and dry.—BURNEY YEO, *A Manual of Medical Treatment.*

